# Muscle fiber characteristics and postmortem quality of *longissimus thoracis*, *psoas major* and *semitendinosus* from Chinese Simmental bulls

**DOI:** 10.1002/fsn3.1898

**Published:** 2020-09-20

**Authors:** Yumiao Lang, Songshan Zhang, Peng Xie, Xiaoxi Yang, Baozhong Sun, Hongru Yang

**Affiliations:** ^1^ Key Laboratory of Public Health Safety of Hebei Province College of Public Health Hebei University Baoding China; ^2^ Institute of Animal Science Chinese Academy of Agricultural Sciences Beijing China

**Keywords:** beef quality, electron microscopy, muscle fiber characteristics, *R* values, tenderness

## Abstract

Using Chinese Simmental cattle *semitendinosus*, *psoas major*, and *longissimus thoracis* samples, we assessed muscle fiber characteristics and postmortem quality. The type I, IIA, and IIB fiber diameters were greater in *semitendinosus* and *longissimus thoracis* relative to *psoas major*, with *psoas major*, *semitendinosus*, and *longissimus thoracis* having the highest respective percentages of type I, IIB, and IIA fibers. *Psoas major* had the highest *R*_248_ and *R*_250_ values and lowest *R*_258_ values at 1‐ and 6‐hr postmortem. *Psoas major* had the lowest Warner–Bratzler shear force (WBSF), hardness, and chewiness values. The trends of WBSF, hardness, and chewiness changes decreased with increasing aging time. *Semitendinosus* had higher changes in WBSF than *psoas major*, and the number % type I fibers was correlated negatively with % changes of WBSF. Therefore, muscles with a high proportion of type IIB fibers and a low proportion of type I had lower tenderness and higher tenderization rate. Further research should be done to seek the optimal composition of muscle fiber type in order to improve beef quality, as muscle fiber type has opposite effect of tenderness background and tenderization rate.

## Graphical abstract

Muscle fiber characteristics and postmortem quality of longissimus thoracis (LT), psoas major (PM), and semitendinosus (ST) from Chinese Simmental cattle were studied. Results showed that muscles with a high proportion of type IIB fibers and a low proportion of type I had lower tenderness and higher tenderization rate.

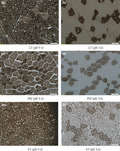

## 1 INTRODUCTION

With the development of economy, individual muscles have been become popular in Chinese market. Chinese consumers are paying more attention to meat quality, which is highly variable due to preslaughter and postslaughter conditions and postmortem aging (Almli, Van Wezemael, Verbeke, & Ueland, 2013; Hwang, Kim, Jeong, Hur, & Joo, 2010; Joo, Kim, Hwang, & Ryu, 2013; Lee, Joo, & Ryu, 2010). Postmortem aging improves beef tenderness (Tatum, Belk, George, & Smith, 1999). However, the postmortem tenderization rate and the time required to achieve the ideal tenderness of individual muscles are different (Belew, Brooks, McKenna, & Savell, 2003; Gruber, Belk, Belk, Tatum, Scanga, & Smith, 2006; Gruber, Tatum, et al., 2006). The knowledge about what factors and how they affect these differences was very important for the industry to improve beef tenderness.

Skeletal muscle is composed by heterogeneous muscle fibers, and beef qualities are closely related to muscle fiber characteristics. Muscle fiber types differ according to their metabolism and contractile response. Muscle fiber type can be divided into type I, IIA, and IIB through the histochemical techniques (ATPase staining method; Brooke & Kaiser, 1970). Four fiber types (I, IIA, IIB, and IIX) can be classified from the detection of myosin heavy chain isoforms (MyHC I, IIa, IIb, and IIx) using immunohistochemical techniques and electrophoretic separation method. However, many studies found that IIB fiber (expressing IIB myosin heavy chain) was not expressed in trunk and limb muscles of bovine (Picard & Gagaoua, 2020).

Muscle fiber characteristics are key factors significantly affecting meat qualities, such as pH decline, tenderness, water‐holding capacity, color parameters (Hwang et al., 2010; Joo, Joo, & Hwang, 2017; Kirchofer, Calkins, & Gwartney, 2002; Van Bibber‐Krueger et al., 2020), connective tissue component, and intramuscular fat content (Normand *et al*., 2020; Listrat, Gagaoua, Normand, Gruffat, et al., 2020). Muscle fiber types significantly affect glycogen and mitochondria contents, determined the glycolytic rate, then influenced myofibrillar protein degradation, enzymatic activity of glycolytic enzymes and calcium‐dependent proteolysis system, and finally affected the ultimate meat quality (Choi & Kim, 2009; Picard & Gagaoua, 2020). Studies showed that total collagen, insoluble collagen, and chemical cross‐links were positively and negatively correlated with type IIA and IIB + X muscle fibers, insoluble collagen, and sensory tenderness was correlated with type I muscle fibers in cattle muscle, regardless of muscle and animal type (Listrat, Gagaoua, Normand, Andueza, et al., 2020; Listrat, Gagaoua, Normand, Gruffat, et al., 2020). Type IIB muscle fibers have higher calpain/calpastatin ratios compared to type I fibers in porcine muscle; myosin heavy chain (MHC) slow isoform contents are positively correlated with calpastatin activity (Choi & Kim, 2009; Ouali & Talmant, 1990). Muscle proteins, for example, desmin and troponin T, of different muscle fiber types have different degradation rates (Christensen, Henckel, & Purslow, 2004; Muroya, Ertbjerg, Pomponio, & Christensen, 2010). Therefore, muscle fiber type influences glycolytic and protein degradation rates, thereby affecting postmortem meat quality. Several studies have focused on how characteristics of muscle fibers impact meat quality (Chang et al., 2003; Hwang et al., 2010; Renand, Picard, Touraille, Berge, & Lepetit, 2001; Zhang et al., 2014); however, there is little information on the effect of muscle fiber characteristics on the postmortem meat quality of Chinese Simmental cattle, especially on beef tenderization rate. This study thus aimed to evaluate how the characteristics of muscle fibers impacted the postmortem meat quality of *longissimus thoracis* (LT), *psoas major* (PM), and *semitendinosus* (ST) from Chinese Simmental bulls in an effort to better understand how these characteristics can be manipulated to modulate postmortem beef quality.

## 2 MATERIALS AND METHODS

### 2.1 Materials

Ten Chinese Simmental cattle (26 month of age, male, carcass weight: 378 ± 30 kg), reared in a grass‐based system supplemented with concentrates, were chosen from a local feedlot and transported to a modern beef slaughterhouse (Beijing Yuxiangyuan livestock co., Ltd). After a 12‐hr fasting and freedom drinking water, animals were randomly slaughtered via Halal approaches. At 30 min following slaughter, LT, PM, and ST were excised from the right carcasses, separated to produce five steaks of 6 cm in thickness, vacuum‐packed, and kept for 1, 3, 7, 14, and 21 days at 4°C. Roughly samples (1 × 1×1 cm cubes) utilized for histochemical analyses were isolated from the centers of LT (sixth rib), PM, and ST muscles within 1‐hr postslaughter, snap‐frozen in liquid nitrogen, and then stored at −80°C (Oury, Dumont, Jurie, Hocquette, & Picard, 2010). In order to cover a wide range of tenderness and muscle fibers composition, these three major muscles were chosen due to their different beef qualities and muscle fiber characteristics (Belew et al., 2003; Kirchofer et al., 2002).

### 2.2 Histochemical analysis

Histochemical analyses were performed with a modified version of a protocol as reported by Brooke and Kaiser (1970). Transverse serial sections (8 μm) were generated with a cryostat (CM1950, Leica Microsystems Nussloch GmbH) and stained for myosin ATPase after preincubation with alkaline (pH 9.4) and acid (pH 4.6). Images of all sections were captured by orthopedic biological microscope (Olympus BX61, Olympus Corporation). About 300 fibers of each sample were analyzed in terms of size and fiber type, that is, I, IIA, or IIB. In this paper, IIB fiber, which was classified by ATPase staining method, was corresponding to IIX fiber (expressing IIX myosin heavy chain isoform). Fiber number and area percentages, as well as fiber diameters (FNP, FAP, and FD, respectively), were established with Image‐Pro Plus 6.0 software (Media Cybernetics). FNP refers to the ratio of counted fiber number of each fiber type to the total counted fiber number. FAP was the ratio of total cross‐sectional area of each fiber type to total fiber area (Hwang, Joo, Bakhsh, Ismail, & Joo, 2017; Joo, Lee, Hwang, & Joo, 2017).

### 2.3 *R* values

Approximately 1 g of muscle samples was homogenized in 0.85 M perchloric acid (12.5 ml) at 3,000 *g* for 90 s (XHF‐D, Xinzhi instrument Co.) and centrifuged (TGL17M, Shanghai Luxiangyi instrument Co.) at 3,000 *g* for 10 min at 2°C. Absorbance at 250, 250, and 260 nm was recorded (Ryu & Kim, 2006). The absorbance ratios of 248 nm:260 nm, 250 nm:260 nm, and 258 nm:250 nm were defined as *R*_248_, *R*_250,_ and *R*_258_, respectively.

### 2.4 Meat quality characteristics

#### 2.4.1 pH

pH was measured at 1, 6, 12, and 24 hr and 3, 7, 14, and 21 days postmortem in the center of the steak of three muscles (Lu, Zhang, Zhu, Luo, & Hopkins, 2018). IQ pH meter (IQ Scientific Instruments, Inc) equipped with a combination electrode was used, and the pH meter was calibrated with pH 4.0 and 7.0 buffers.

#### 2.4.2 Tenderness

Warner–Bratzler shear force (WBSF) was assessed according to Li et al. (2006). Steaks were cooked at 80°C in a water bath to 70°C internal temperature. Temperature was monitored with a testo 205 (Testo Instrument Company). Six beef cubes (1 × 1 × 3 cm), having the longest axis (3 cm) parallel to the fiber direction, were obtained, and WBSF was determined by texture analyzer (TA.XT Plus, Stable Micro System, Ltd) using the HDP/BSW blade.

Texture profile analysis was conducted according to Leick et al. (2012) using the texture analyzer. Six 1 × 1 × 1 cm cubes of cooked beef steaks were subjected to 2 75% compression cycles via P/75 probe. Textural parameters (chewiness, hardness, adhesiveness, cohesiveness, gumminess, springiness, and resilience) were determined as described by Caine, Aalhus, Best, Dugan, and Jeremiah (2003) and Leick et al. (2012).

#### 2.4.3 Pressing loss and cooking loss

Pressing and cooking loss were established based on approaches reported by Li, Zhang, et al. (2012)) and Li, Liu, et al. (2012). Cooking loss was measured from steaks prepared for WBSF measurements and given as percentage weight loss prior to and following completion of cooking.

#### 2.4.4 Color

Meat color was analyzed after exposing freshly cut surface to atmospheric air at 4°C for 30 min. Meat color was taken with a CR‐400 chroma meter (Minolta Co., Osaka, Japan) using illuminant D65 for L* (light), a* (red), and b* (yellow) (Cho et al., 2010). The instrument was precalibrated on a white plate according to the guidelines of the manufacturer.

### 2.5 Electron microscopy

Samples were prefixed in 2.5% glutaraldehyde and then fixed with 1% OsO4, followed by ethanol dehydration and spur resin embedding. A Leica UC6 ultramicrotome was used to prepare sections that were stained using uranyl acetate as well as lead citrate (Lang et al., 2016). And the images were taken using a transmission electron microscope (Hitachi H‐7500). The sarcomere length was assessed with Image‐Pro Plus 6.0 software (Media Cybernetics).

### 2.6 Statistical analysis

SAS v9.1 was used for statistical assessment. ANOVA and Duncan’s multiple range test were utilized for comparing the mean values of muscle characteristics and meat quality at a 5% level of significance. Furthermore, percentage changes from days 1–21 postmortem was determined for WBSF as follows: % change WBSF = (WBSF_1d_ – WBSF_21d_)/WBSF_1d_ × 100%, and was defined as tenderization rate (Gruber, Belk, et al., 2006; Gruber, Tatum, et al., 2006; Li, Liu, et al., 2012; Li, Zhang, et al., 2012). Spearman correlation coefficients were used to describe the relationship between WBSF and muscle fiber characteristics.

## 3 RESULTS AND DISCUSSION

### 3.1 Characteristics of muscle fibers

**Figure 1:**
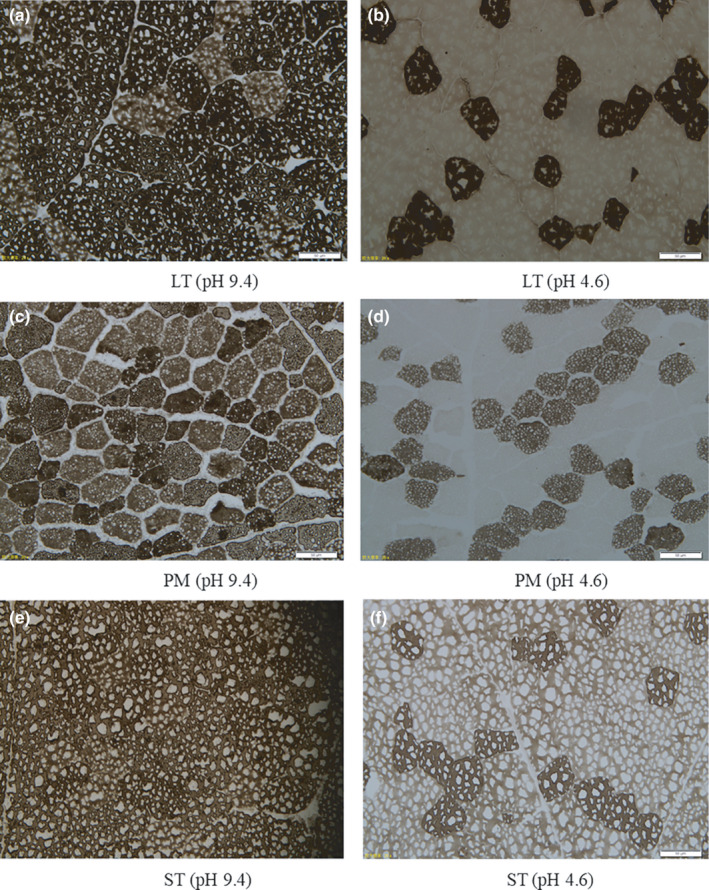
Serial sections of LT, PM, and ST, stained for actomyosin Ca^2+^ ATPase reactivity after preincubation at pH 9.45 and pH 4.63 (bar = 50 μm). ST: *semitendinosus*; LT: *longissimus thoracis*; PM: *psoas major*. The length of the white scale bar is 50 μm

Muscle type had significant effects on muscle fiber diameter and composition (*p* < .05), as shown in Table 1 and Figure 1. In order of decreasing FD, the muscle fiber types were IIB > IIA > I. FD of type I, IIA, and IIB fibers in ST and LT was bigger relative to PM (*p* < .05), and this was in line with the study by Kim, Yang, and Jeong (2016) and Joo, Joo, et al. (2017). FNP of type I was the highest in PM and the lowest in ST (*p* < .05). FNP of type IIA was higher in LT than PM (*p* < .05); no differences were obtained in FNP of type IIA between LT and ST or between PM and ST (*p* > .05). FNP of type IIB was the highest in ST and the lowest in PM (*p* < .05). LT had an increased FAP of type IIA than PM and ST (*p* < .05). PM had a higher FAP of type I, but had a lower FAP of type IIB (*p* < .05). ST had a lower FAP of type I and IIA, but had a higher FAP of type IIB (*p* < .05).

**Table 1:** Muscle fiber characteristics of Chinese Simmental cattle PM, LT, and ST muscles

	LT	PM	ST
FD/μm			
I	35.47 ± 0.66^a^	22.24 ± 3.03^b^	38.68 ± 1.13^a^
IIA	46.05 ± 1.43^a^	26.19 ± 3.29^b^	48.54 ± 1.84^a^
IIB	49.78 ± 1.35^a^	30.62 ± 3.31^b^	52.33 ± 1.75^a^
FNP/%			
I	33.09 ± 1.15^b^	47.32 ± 1.70^a^	22.67 ± 1.89^c^
IIA	27.41 ± 1.54^a^	21.91 ± 1.41^b^	22.96 ± 1.70^ab^
IIB	39.51 ± 1.52^b^	30.56 ± 2.16^c^	54.38 ± 2.25^a^
FAP/%			
I	23.09 ± 1.66^b^	39.01 ± 2.17^a^	16.12 ± 1.80^c^
IIA	31.51 ± 1.82^a^	25.91 ± 1.62^b^	24.92 ± 1.86^b^
IIB	45.41 ± 1.84^b^	35.07 ± 2.28^c^	58.96 ± 2.28^a^

Note

Values are expressed as means ± standard error (*SE*); a–c: different letters represent significant differences within a given row (*p* < .05).

FAP, fiber area percentage; FD, fiber diameter; FNP, fiber number percentage; LT, *longissimus thoracis*; PM, *psoas major*; ST, *semitendinosus*.

Our results showed that FAP and FNP were different among LT, PM, and ST, and this was in line with prior work. Hwang et al. (2017) found PM to exhibit the highest and lowest FNPs for type I and type IIB, respectively, while *semimembranosus* (SM) exhibited maximal type IIB FNP in Korean Native Black Goat. Hwang et al. (2010) reported that type IIA and IIB FNP and FAP were lower in SM muscle, but elevated in PM muscle than LT and SM in Hanwoo steers.

### 3.2 *R* values

*R* values, which reflect the muscle metabolism, are related to the ratio of the relative concentrations of the adenine nucleotides to inosine monophosphate and inosine (Calkins, Dutson, Smith, & Carpenter, 1982; Ryu & Kim, 2005). As shown in Table 2, muscle type and aging had significant effects on *R*_250_, *R*_248,_ and *R*_258_ (*p* < .001). The order of *R*_248_ and *R*_250_ in LT were 24 hr and 72 hr > 12 hr > 6 hr > 1‐hr postmortem. The order of *R*_258_ in LT was 12 hr, 24 hr, and 72 hr < 6 hr < 1‐hr postmortem. *R*_248_ and *R*_250_ in PM at 1‐hr postmortem were significant lower than those at 6‐hr and 24‐hr postmortem (*p* < .05). *R*_248_ and *R*_250_ in ST at 1‐hr postmortem were lower than those at 12‐hr, 24‐hr, and 72‐hr postmortem (*p* < .05). *R*_258_ in ST at 1‐hr postmortem was higher than this at 24‐hr and 72‐hr postmortem (*p* < .05). At 1‐hr postmortem, PM had higher *R*_248_ than LT and ST (*p* < .05); PM had the highest *R*_250_ and LT had the lowest *R*_250_; and *R*_258_ was the highest in LT and the lowest in PM. At 6‐hr postmortem, *R*_248_ and *R*_250_ were the highest in PM and the lowest in LT; *R*_258_ was higher in LT than in ST; and *R*_258_ was higher in ST than in PM (*p* < .05). Results showed that metabolic rate of PM was faster than LT and ST, and this was consistent with the former study by Lefaucheur (2010) showing more rapid pH decline in pork PM relative to *Semispinalis* and *Longissimus*. This may be correlated with higher FNP of type I fibers in PM, and type I fibers had rapid metabolism rate in the sarcoplasm (Cassens & Newbold, 1967; Melody et al., 2004).

**Table 2:** *R* values of LT, PM, and ST in Chinese Simmental cattle during postmortem aging time

	Aging time				
	1 hr	6 hr	12 hr	24 hr	72 hr
*R*_248_					
LT	0.81^dB^	0.94^cC^	1.18^b^	1.32^a^	1.30^a^
PM	1.27^bA^	1.36^aA^	1.32^ab^	1.36^a^	1.33^ab^
ST	0.98^bB^	1.16^abB^	1.23^a^	1.39^a^	1.36^a^
*R*_250_					
LT	0.85^dC^	0.98^cC^	1.20^b^	1.32^a^	1.30^a^
PM	1.28^bA^	1.36^aA^	1.34^ab^	1.36^a^	1.34^ab^
ST	1.02^bB^	1.17^abB^	1.24^a^	1.39^a^	1.36^a^
*R*_258_					
LT	1.18^aA^	1.05^bA^	0.88^c^	0.81^c^	0.81^c^
PM	0.81^C^	0.80^C^	0.81	0.80	0.81
ST	1.02^aB^	0.92^abB^	0.87^ab^	0.78^b^	0.80^b^

Note

a–c: Different letters correspond to differences in a given row that are significant (*p* < .05); A–C: different letters correspond to differences in a given column that are significant (*p* < .05).

LT, *longissimus thoracis*; PM, *psoas major*; ST, *semitendinosus*.

### 3.3 Meat quality characteristics

#### 3.3.1 pH

**Figure 2:**
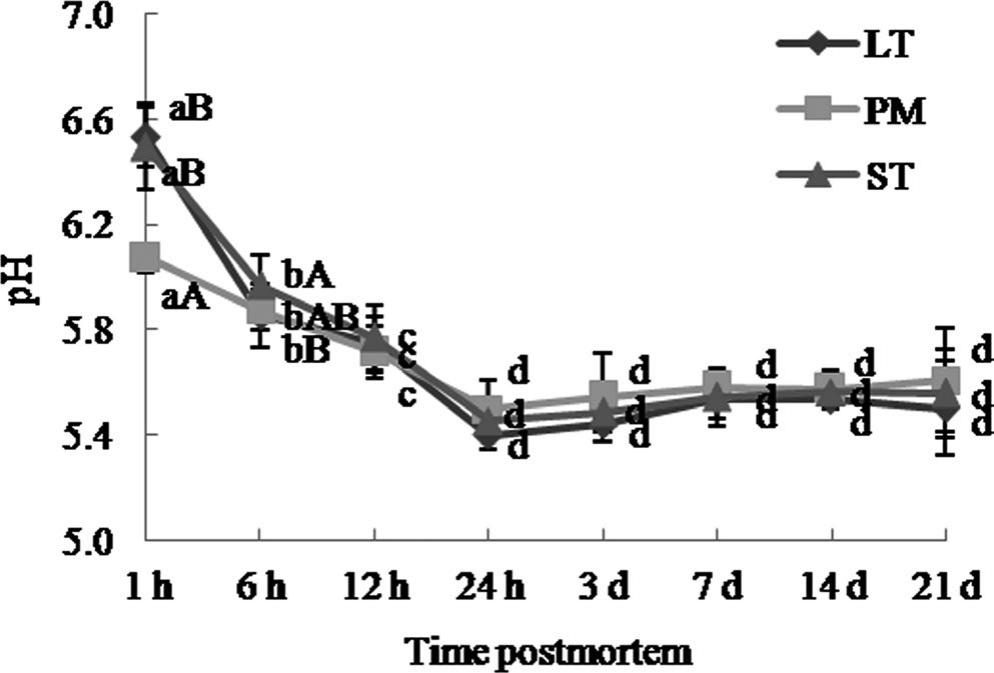
pH decline in three major muscles in Chinese Simmental cattle during postmortem aging time. ST: *semitendinosus*; LT: *longissimus thoracis*; PM: *psoas major*. a–d: Different letters correspond to differences in different postmortem time that are significant (*p* < .05); A–B: different letters correspond to differences in muscle types that are significant (*p* < .05)

The pH decline of LT, PM, and ST during postmortem aging was determined (Figure 2). During the first 24‐hr postmortem, pH decreased and then reached a plateau at 24 hr. At 1 hr, pH values of PM were statistically lower than that of LT and ST (*p* < .05). At 6‐hr postmortem, ST had higher pH values than LT (*p* < .05), in line with the former study by Wang et al. (2018). From 12‐hr to 21‐day postmortem, pH values among LT, PM, and ST did not differ significantly (*p* > .05). The result suggested that PM had a higher pH decline rate following slaughter, and this was in agreement with the *R* values and the literature that red muscles had higher rate and extent of pH decline than meat with more percentages of fast‐twitch glycolytic fibers (Picard & Gagaoua, 2020).

#### 3.3.2 Tenderness

**Figure 3:**
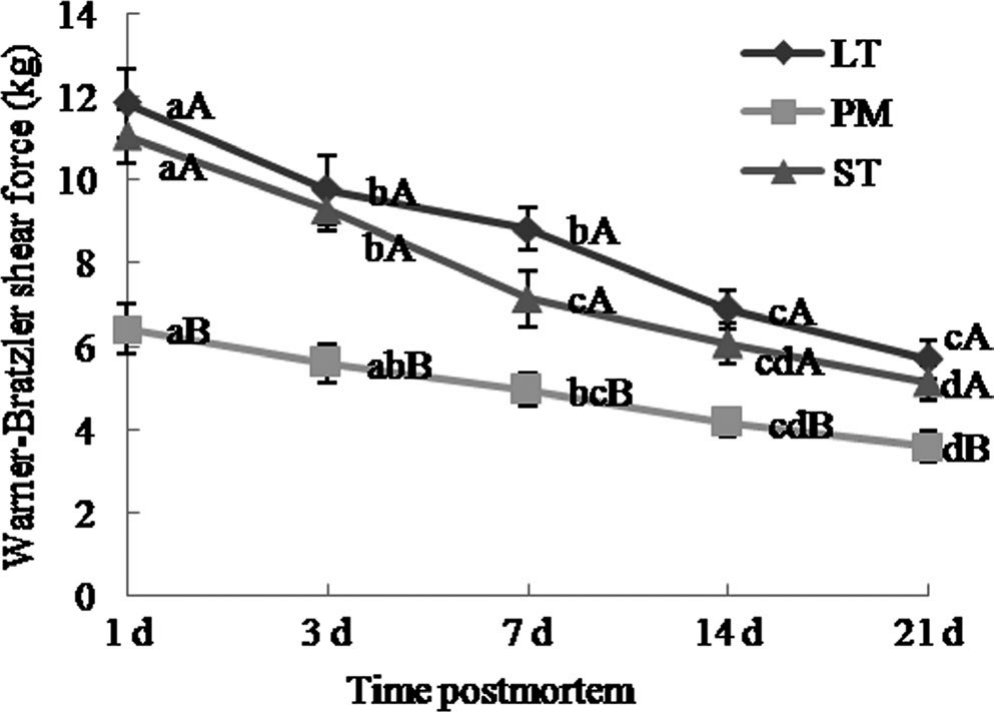
Warner–Bratzler shear force (WBSF) of Chinese Simmental cattle ST, LT, and PM during postmortem aging time. ST: *semitendinosus*; LT: *longissimus thoracis*; PM: *psoas major*. a–d: Different letters correspond to differences in different postmortem time that are significant (*p* < .05); A–B: different letters correspond to differences in muscle types that are significant (*p* < .05)

The WBSF changes during postmortem aging time are shown in Figure 3. Muscle type and aging time had significant effects on WBSF (*p* < .01). During 21‐day postmortem, ST and LT exhibited higher WBSF than PM (*p* < .05). WBSF decreased with increasing aging time, the trend of WBSF changes was decreasing (*p* < .05). ST had higher changes in WBSF than PM (*p* < .05, Table 3). The relationship between myofiber characteristics and tenderization rate was analyzed (Table 4), and results showed that fiber diameter was positively correlated with WBSF changes (*p* < .05). FNP of type IIB correlated positively with WBSF changes (*r* = .378; *p* < .05). FAP of type I fibers was correlated negatively with WBSF changes, and FAP of type IIB was correlated positively with WBSF changes (*r* = .383; *p* < .05). FNP of type I was correlated negatively with % changes of WBSF (*r* = −.303; *p* < .05).

**Table 3:** The ΔWBSF and % change WBSF of Chinese Simmental cattle LT, PM, and ST

	ΔWBSF (kg)	% change WBSF
LT	6.11 ± 0.56^a^	51.54 ± 2.47^ab^
PM	2.94 ± 0.51^b^	43.69 ± 4.04^b^
ST	5.94 ± 0.52^a^	53.52 ± 8.25^a^

Note

Values are expressed as means ± standard error (*SE*); a–b: different letters correspond to significant differences in a given column (*p* < .05).

ΔWBSF = change from days 1 to 21 for Warner–Bratzler shear force; % change WBSF = (WBSF_1d_ – WBSF_21d_)/WBSF_1d_ × 100%; LT, *longissimus thoracis*; PM, *psoas major*; ST, *semitendinosus*.

**Table 4:** The correlation analysis of muscle fiber characteristics and tenderization rate

	ΔWBSF	%change WBSF
FD		
I	0.535**	0.296
IIA	0.528**	0.330
IIB	0.522**	0.275
FNP		
I	−0.346	−0.303*
IIA	0.248	0.222
IIB	0.378*	0.187
FAP		
I	−0.474*	−0.334
IIA	0.138	0.202
IIB	0.383*	0.216

Note

ΔWBSF = change from days 1 to 21 for Warner–Bratzler shear force; %change WBSF = (WBSF_1d_ – WBSF_21d_)/WBSF_1d_ × 100%; FAP, fiber area percentage; FD, fiber diameter; FNP, fiber number percentage.

* *p* < .05;

** *p* < .01.

**Figure 4:**
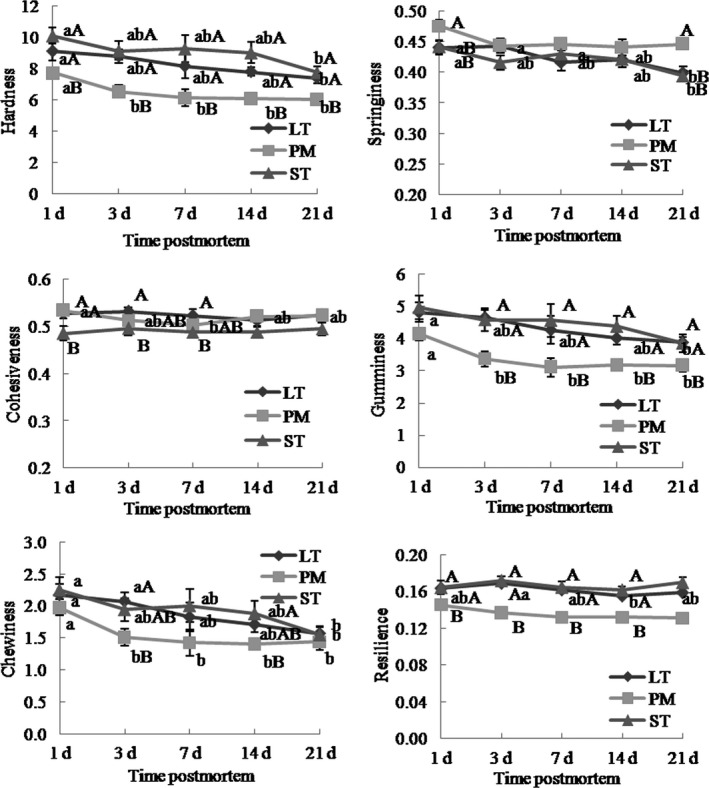
Texture profile analysis (TPA) of Chinese Simmental cattle ST, LT, and PM during postmortem aging time. ST: *semitendinosus*; LT: *longissimus thoracis*; PM: *psoas major*. a–b: Different letters correspond to differences in different postmortem time that are significant (*p* < .05); A–B: different letters correspond to differences in muscle types that are significant (*p* < .05)

Aging time had significantly impacted gumminess, hardness, springiness, and chewiness (*p* < .01), but not on cohesiveness or resilience (*p* > .05), as shown in Figure 4. With increasing aging time, the trends of hardness and chewiness changes in LT, PM, and ST were decreasing, the trends of springiness changes in LT and ST were decreasing, and the trends of gumminess in LT and PM decreased. Muscle type had significant effects on springiness, chewiness, cohesiveness, hardness, gumminess, and resilience (*p* < .001). Hardness and resilience were lower in PM than in LT and ST during the 21 days (*p* < .05). Springiness was higher in PM than in LT and ST at 1 and 21 days (*p* < .05). Cohesiveness was higher in LT than in ST at 1, 3, and 7 days (*p* < .05). Gumminess was higher in LT and ST than in PM at 3, 7, 14, and 21 days (*p* < .05). Chewiness was the lowest in PM at 3 and 14 days (*p* < .05).

Our study shows that PM muscle had lower WBSF, hardness, and chewiness values than LT and ST muscle, and this was consistent in line with work from Kim et al. (2016) and Rodriguez et al. (2014). The results may be partly explained muscle fiber composition and fiber size of these three major muscles. Some former studies had shown that tenderness was higher for muscle with high frequency of type I and lower frequency of type IIB fibers. Muscles with larger fiber area had greater hardness than muscle with smaller fiber size, particularly type IIB fiber (Renand et al., 2001; Żochowska et al., 2005). And this was consistent with our study that PM had higher percentage of type I fibers and also small fiber size. However, some studies have reported that muscles with increased type I fiber frequencies and a low proportion of type IIB fibers have higher WBSF values (Chriki et al., 2013; Ozawa et al., 2000; Ryu & Kim, 2005). The contradictory results may be explained by breeds, muscle type, and muscle location used in the experiments (Jurie et al., 2007; Maltin, Balcerzak, Tilley, & Delday, 2003; Oury et al., 2010; Van Bibber‐Krueger et al., 2020).

In this study, we first described the relationship between muscle fiber type and % changes of WBSF. PM had fewer changes in WBSF, ST had higher changes in WBSF, and FNP of type I was negatively correlated with % changes of WBSF. And this was consistent with the former study that type I fibers have lower calpain/calpastatin ratios than type IIB fibers (Ouali & Talmant, 1990). Moreover, muscle fiber types have different susceptibility to protein denaturation, especially desmin and troponin T (Bowker, Swartz, Grant, & Gerrard, 2005; Christensen et al., 2004). Type IIB fibers have thinner Z‐band than type I fibers; proteins comprising Z‐bands in type IIB fibers are more vulnerable to rapid postmortem proteolytic breakdown compared with those of type I fibers (Muroya et al., 2010).

#### 3.3.3 Pressing loss and cooking loss

**Figure 5:**
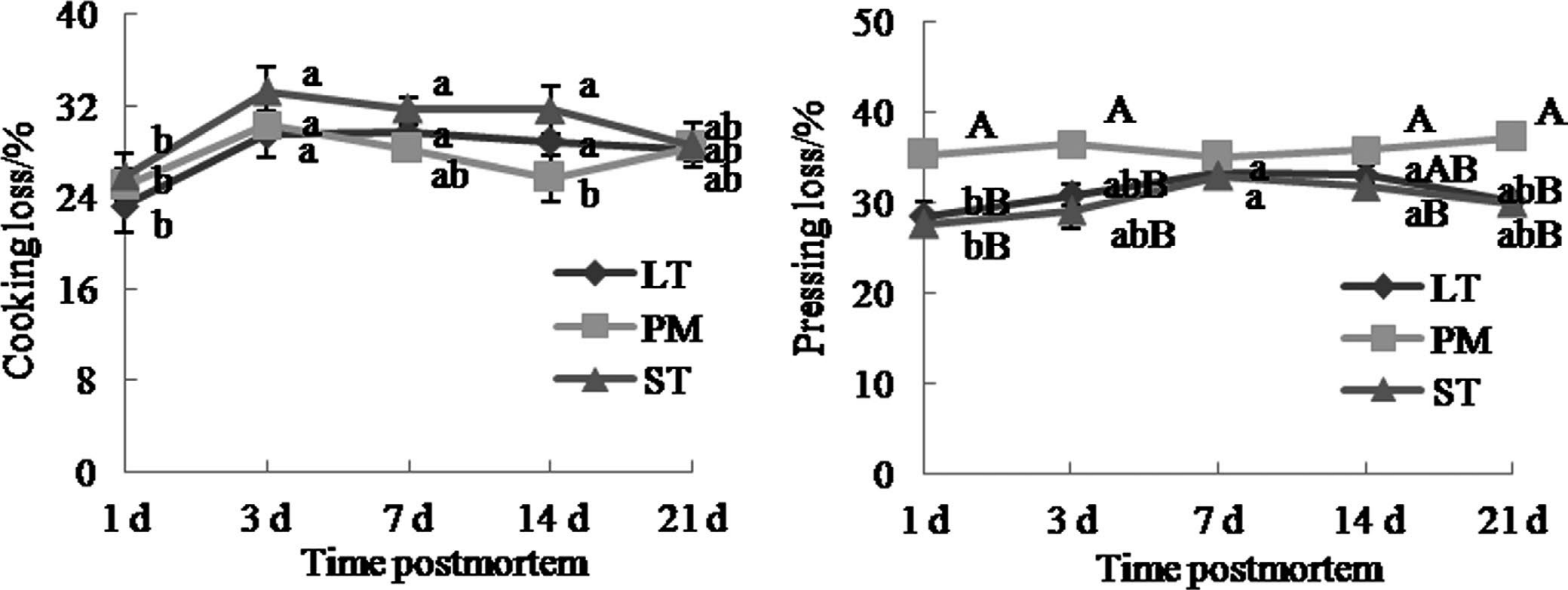
Pressing loss and cooking loss of Chinese Simmental cattle ST, LT, and PM during postmortem aging time. ST: *semitendinosus*; LT: *longissimus thoracis*; PM: *psoas major*. a–b: Different letters correspond to differences in different postmortem time that are significant (*p* < .05); A–B: different letters correspond to differences in muscle types that are significant (*p* < .05)

Muscle type did not impact cooking loss (*p* > .05, Figure 5), but it had significant effects on pressing loss (*p* < .05). At 1, 3, and 21 days after death, ST and LT exhibited less pressing loss than did PM (*p* < .05). At 14‐day postmortem, pressing loss in ST was lower than that in PM (*p* < .05); pressing loss did not differ between ST and LT (*p* > .05). Therefore, PM had a lower water‐holding capacity than LT and ST. Aging time had significant effects on cooking loss and pressing loss (*p* < .01). Cooking loss of LT, PM, and ST increased from 1‐ to 3‐day postmortem (*p* < .05), and the trends of cooking loss changes were not obvious from 3‐ to 21‐day postmortem (*p* > .05). PM pressing loss during 21‐day postmortem did not differ significantly (*p* > .05). Pressing loss in LT and ST at 7‐ and 14‐day postmortem were higher than that at 1‐day postmortem (*p* < .05), but had no differences from that at 3‐ and 21‐day postmortem (*p* > .05).

Results showed that PM had a lower water‐holding capacity than ST and LT. In line with past results, there was a negative correlation between myofiber cross‐sectional area and drip loss and thawing loss (Berri et al., 2007; Kim et al., 2008), and this was consistent with our study that the diameter of PM was lower in PM than this in LT and ST. However, some studies showed that early postmortem drip loss was greater when muscles contained fewer type I fibers and more type IIB fibers (Choe et al., 2008; Lefaucheur, 2010). These differences may be associated with muscle physiology, buffering capacity, and pH (Lee et al., 2010). And the fast pH decline causes autolysis of enzyme, denaturation of sarcoplasmic and myofibrillar protein, resulting in a decrease in the ability to hold water (Pomponio, Ertbjerg, Karlsson, Costa, & Lametsch, 2010; Scheffler & Gerrard, 2007).

#### 3.3.4 Color parameters

**Figure 6:**
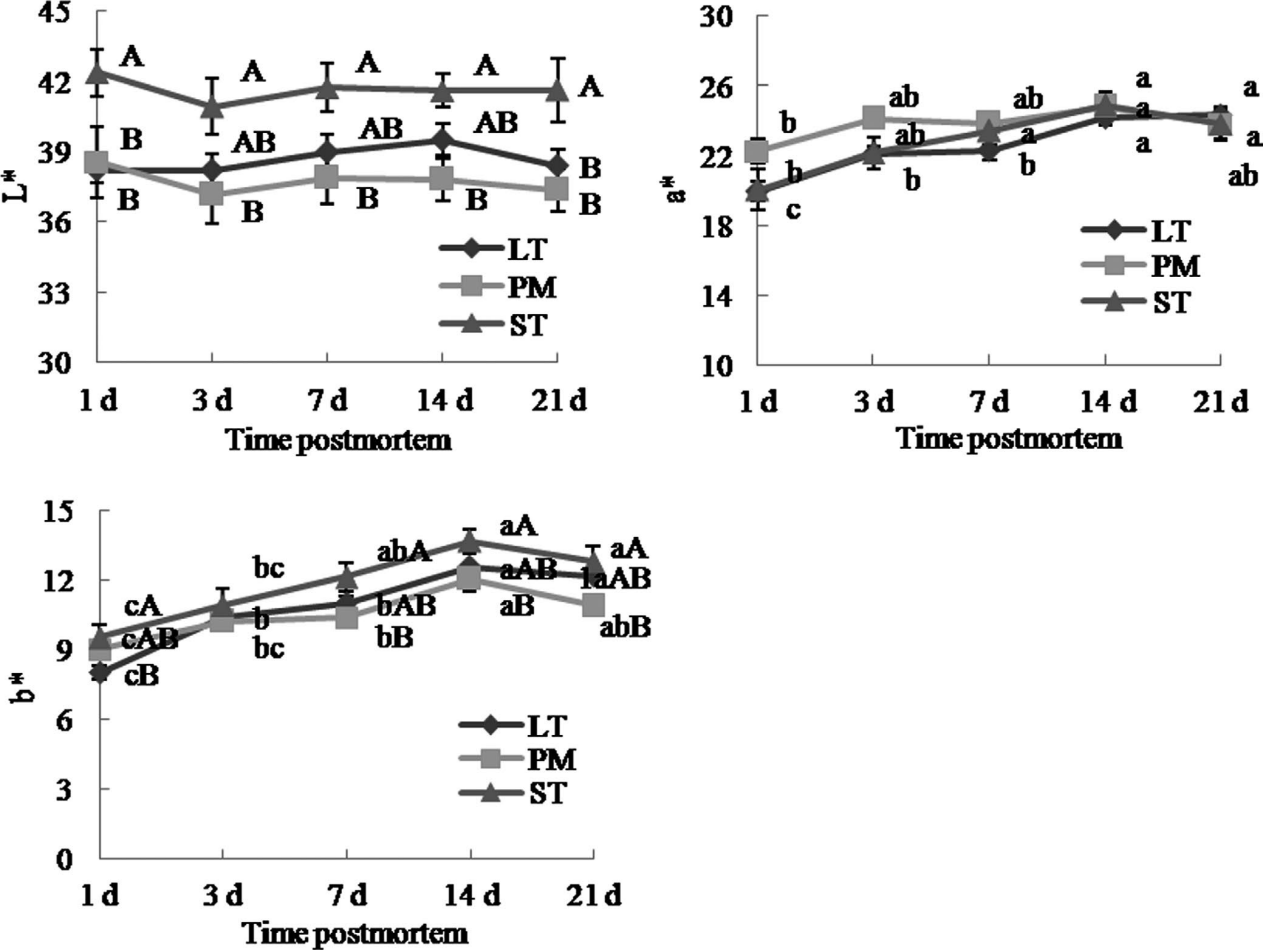
Beef color (L*, a*, and b*) of Chinese Simmental cattle ST, LT, and PM during postmortem aging time. ST: *semitendinosus*; LT: *longissimus thoracis*; PM: *psoas major*. a–b: Different letters correspond to differences in different postmortem time that are significant (*p* < .05); A–B: different letters correspond to differences in muscle types that are significant (*p* < .05)

Muscle type had significant impacts on color parameters (*p* < .05, Figure 6). At 1 and 21 days, L* of ST was greater than that of LT and PM (*p* < .05). At 3, 7, and 14 days, L* of ST was higher than that of PM (*p* < .05). The a* values in PM were higher than that in LT and ST at 1 and 3 days, but there was no statistic difference. At 1 day, b* of ST was higher than that of LT (*p* < .05). At 7, 14, and 21 days, ST had higher b* values than PM (*p* < .05). Aging time had significant effects on a* and b* (*p* < .05), but not on L* (*p* < .05). With increasing aging time, the trends of a* and b* values changes in LT, PM, and ST were increasing. Result showed that ST had higher L* and b* values, and PM had higher a* values. Some studies reported that muscle fiber type was closely related with color parameters and muscles with lower percentage of type I fibers showed lower a* values and higher L* values (Choe et al., 2008; Kim et al., 2013), and this was consistent with our study.

### 3.4 Electron microscopy

**Figure 7:**
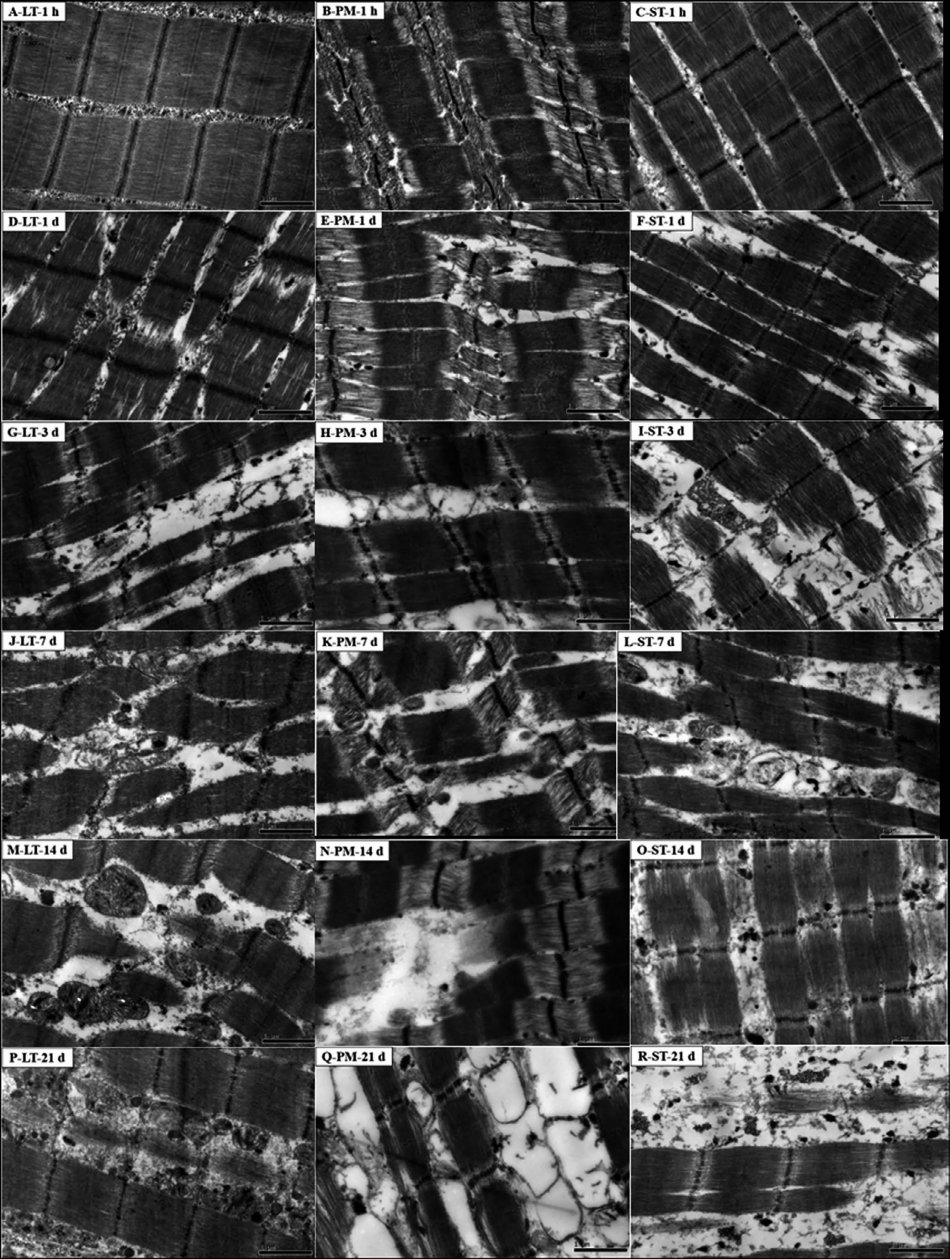
Electron micrographs of LT, PM, and ST during postmortem aging period. ST: *semitendinosus*; LT: *longissimus thoracis*; PM: *psoas major*. The length of the black scale bar is 1 μm

Electron microscopy of LT, PM, and ST was shown in Figure 7. At 1‐hr postmortem, the sarcomere length of PM was longer greatly, due to the I band of PM was weak. At 1‐day postmortem, myofibril structure was disrupted, I band breakage appeared in both PM and ST muscles, while M‐lines in LT and ST became obscured even disappeared. At 3‐day postmortem, boundaries between the A and I bands and the H region were visibly weaker among LT, PM, and ST muscles. As the aging time increased, myofibrils ruptured and fragmented. Results show that muscle type affects myofibrils degradation and the degradation of myofibril proteins in PM was faster than LT and ST. This was consistent with the former study that calpain can be activated earlier, due to increased sarcoplasmic Ca^2+^ concentration in PM muscle (Melody et al., 2004). Calpains are widely considered as the major endogenous enzyme in postmortem proteolysis of myofibrillar proteins (Ding et al., 2018), and the earlier activation of μ‐calpain may contribute to the higher rate of tenderization (Yan et al., 2018).

## 4 CONCLUSION

This study is the first to describe the relationship between muscle fibers composition and tenderization rate. Muscle type had significant effects on muscle fiber composition, *R* values, and overall qualities of meat, especially tenderness. Relative to LT and ST, PM muscle exhibited a higher rate of pH decline, *R*_248_ and *R*_250_ values. PM muscle, with type I fibers being more prevalent and type IIB fibers being present in lower proportions, had higher tenderness and lower tenderization rate. ST muscle, which has higher type IIB fiber proportions and reduced type I fiber proportions, had lower tenderness and faster tenderization rate. Therefore, muscle with high meat quality should have the optimization of muscle fiber type composition in order to balance the tenderness background and its tenderization rate. Further research should be done to seek the optimal composition of muscle fiber type, as muscle fiber type has opposite effect of tenderness background and tenderization rate.

## CONFLICT OF INTEREST

The authors declare that they have no conflict of interest in this work.
